# Discovery of a High-Efficient Algicidal Bacterium against *Microcystis aeruginosa* Based on Examinations toward Culture Strains and Natural Bloom Samples

**DOI:** 10.3390/toxins15030220

**Published:** 2023-03-14

**Authors:** He Zhang, Yan Xie, Rongzhen Zhang, Zhongliang Zhang, Xinglong Hu, Yao Cheng, Ruozhen Geng, Zengling Ma, Renhui Li

**Affiliations:** 1 Zhejiang Provincial Key Laboratory for Subtropical Water Environment and Marine Biological Resources Protection, National and Local Joint Engineering Research Center of Ecological Treatment Technology for Urban Water Pollution, College of Life and Environmental Sciences, Wenzhou University, Wenzhou 325035, China; 2 State Key Laboratory of Biocatalysis and Enzyme Engineering, School of Life Sciences, Hubei University, Wuhan 430062, China; 3 Wenzhou Shanxi Hydro-junction Management Center, Wenzhou 325035, China; 4 Institute for Eco-Environmental Research of Sanyang Wetland, Wenzhou University, Wenzhou 325035, China

**Keywords:** harmful cyanobacterial blooms, algicidal bacteria, *Microcystis aeruginosa*, microcystins, *Streptomyces*

## Abstract

Harmful cyanobacterial blooms occur worldwide and pose a great threat to aquatic ecosystems and public health. The application of algicidal bacteria represents an eco-friendly strategy for controlling harmful cyanobacterial blooms; thus, searching for a high efficiency of algicidal bacteria has been becoming an important and continuous task in science. Herein, we identified a bacterial strain coded *Streptomyces* sp. HY with a highly algicidal activity, and investigated its algicidal efficiency and mechanism against *Microcystis aeruginosa*. The strain HY displayed high algicidal activity toward *Microcystis aeruginosa* cells, with a removal rate of 93.04% within 2 days via indirect attack. *Streptomyces* sp. HY also showed the ability to lyse several genera of cyanobacterial strains, including *Dolichospermum*, *Pseudanabaena*, *Anabaena*, and *Synechocystis*, whereas it showed a minor impact on the green alga *Scenedesmus obliquus*, demonstrating its selectivity specially for targeting cyanobacteria. Its algicidal mechanism involved damages to the photosynthesis system, morphological injury of algal cells, oxidative stress, and dysfunction of the DNA repair system. Furthermore, HY treatment reduced the expression levels of genes (*mcyB* and *mcyD*) related to microcystin biosynthesis and decreased the total content of microcystin-leucine-arginine by 79.18%. Collectively, these findings suggested that the algicidal bacteria HY is a promising candidate for harmful cyanobacterial bloom control.

## 1. Introduction

Harmful cyanobacterial blooms (HCBs) have become a worldwide concern for water eutrophication and global warming in recent years [1]. More than 60% of the lakes in China are eutrophic and suffer from HCBs, such as Lake Taihu [2]. HCBs can seriously damage aquatic ecosystems and pose a great threat to human health by producing various cyanotoxins. *Microcystis aeruginosa* (*M*. *aeruginosa*) is one of the most dominant harmful cyanobacterial species, as well as the major species producing and releasing microcystins (MCs) worldwide [1]. MCs are a group of cyclic heptapeptides comprising hundreds of variants, and MC-leucine-arginine (MC-LR) is one of the most toxic and abundant variants [3]. Accumulated reports have proved that MC-LR causes liver cancer and tumors via inhibiting the activities of protein phosphatases 1A and 2A in humans and animals [4]. Moreover, MC-LR also displays reproductive toxicity, neurotoxicity, immunotoxicity, and other toxicities [5].

Given that the guide concentration of MC-LR for drinking water is less than 1 μg/L [6], it is urgent to develop cost-effective and eco-friendly approaches to control HCBs caused by *M. aeruginosa*. Several strategies have been proposed to solve HCBs, including chemical, physical, and biological methods [7]. Mechanical removal, adsorption-flocculation, and ultra-sonication are frequently used physical methods. Although these methods are sometimes effective, they are limited by their high costs and low efficiency in large areas and volumes. Chemical methods have been successfully used to control HCBs, with CuSO_4_ [8] and hydrogen peroxide (H_2_O_2_) [9] once popular chemical algicides. However, these agents are non-specific and come with the potential risk of secondary pollution because of their toxicity and fast release of cyanotoxins. These disadvantages greatly limit their general application, and alternative biological tools, especially algicidal bacteria, have attracted considerable attention for their low-cost, eco-friendliness, and great potential [10]. Previous reports have found that many algicidal bacteria not only disrupt the photosystem of certain cyanobacteria, but also suppress the expression of several key genes related to photosystem, significantly decreasing the maximum quantum yield of photosystem II (PSII) and the photosynthetic electron transport rate (rETR) of cyanobacteria [11]. Furthermore, several algicidal strains can also inhibit the expression of genes related to MC biosynthesis [12], even degrading MCs [13]. To date, a number of algicidal bacterial species from about 50 genera have been identified [10,14], including *Bacillus* spp. [15], *Pseudomonas* spp. [16], *Aeromonas* spp. [17], *Raoultella* spp. [11], and *Streptomyces* spp. [18]. *Streptomyces* species are known for their ability to biosynthesize different kinds of secondary metabolites, including antibiotics and other active substances [19].

In general, algicidal bacteria achieve their activities through two mechanisms: (1) direct algicidal activities through cell-to-cell contact with cyanobacterial cells; and (2) indirect algicidal activities by secreting extracellular algicides, such as proteins, antibiotics, pigments, alkaloids, and other substances to kill cyanobacteria [20]. Approximately 30% of bacteria reported are suggested to be direct algicidal bacteria, whereas the remaining 70% are indirect ones [20]. Algicidal bacteria displaying both mechanisms have been rarely identified [21]. Both modes of algicidal bacteria and their active algicidal chemicals are considered as effective agents for controlling HCBs. Another enormous challenge comes from the fact that algicidal bacteria kill *Microcystis* cells and result in fast release of intracellular MCs into the water in a few hours. MCs can remain stable in water for several days, resulting in extensive exposure of all aquatic organisms to MCs before they are completely degraded [6]. Therefore, controlling *Microcystis* blooms and inhibiting MC biosynthesis at the same time through indirect algicidal bacteria is a promising strategy. The major objective of the present work was to isolate and identify novel algicidal bacteria against HCBs, hopefully with higher efficiency than those found in previous studies.

In this study, we investigated a newly isolated algicidal strain (*Streptomyces* sp. HY) from the Dongzhen Reservoir, Putian, Fujian Province. The algicidal process and underlying mechanisms of action against *M. aeruginosa* FACHB 905 were studied. The strain HY strongly inhibited the growth and biosynthesis of MC-LR in *M. aeruginosa* via secreting active compound(s). HY cell-free filtrate induced serious photosystem impairment by dramatically reducing photosynthetic efficiency (F_v_/F_m_) and inhibiting the maximum photosynthetic rate (ETR_max_). After exposure to the HY filtrate, the level of reactive oxygen species (ROS) and content of malondialdehyde (MDA) increased obviously, whereas a number of key genes were downregulated. This study enriched our knowledge of interactions between *Microcystis*-bacteria, and contributed to the development of novel biocontrol approaches against HCBs.

## 2. Results

### 2.1. Isolation and Identification of Algicidal Bacteria

**Figure 1:**
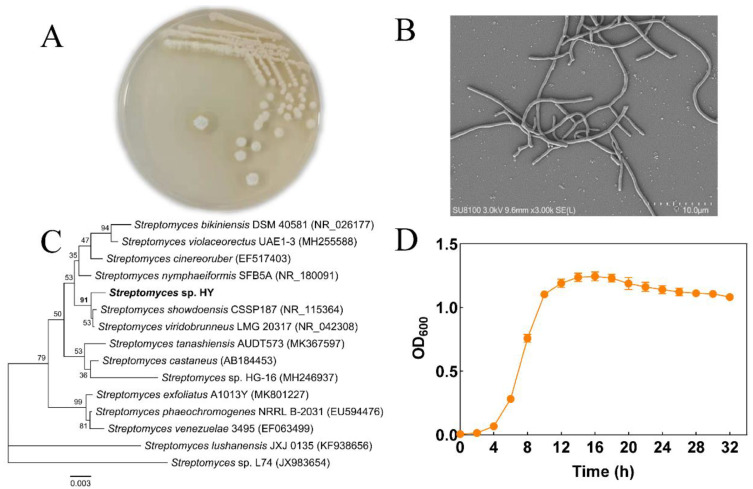
Identification of strain #332 (HY) by morphology and 16S rRNA gene. Colonies on R2A plates at 4 d observed by inverted optical microscope (**A**) and mycelia observed by SEM (**B**). Phylogenetic tree of strain HY based on its 16S ribosomal RNA gene sequence (**C**). The growth curve of HY in R2A liquid medium (**D**).

In total, 411 bacterial strains were isolated during October to December in 2021 from Fujian and Zhejiang Provinces in China, and subsequently tested for their algal activity against *M. aeruginosa* FCHAB 905. Strain #320 from Dongzhen Reservoir (Putian, Fujian, China) exhibited high algicidal activity (Figure 1) and was chosen for further study. The 16S rRNA sequence of this strain shared high similarity (>98.9%) to strains of *Streptomyces* spp. (Figure 1C). In a phylogenetic tree, strain #320 clustered closely with *Streptomyces* spp. strains, although it formed an independent branch. *Streptomyces* sp. #320 grew well on R2A agar medium (Figure 1), as well as on Gause No. 1 agar plates (data not shown), a selective medium for actinomycetes [22]. When incubated for more than 4 days on a plate, colonies were circular with white aerial mycelium, and a mound of grey, rectiflexibilis-type spores were formed (Figure 1A,B). Thus, based on the 16S rRNA sequence analysis and cellular morphology characteristics, this algicidal strain was identified as *Streptomyces* sp. and designated as strain *Streptomyces* sp. HY hereafter.

### 2.2. Algicidal Characteristics of Streptomyces sp. HY

**Figure 2:**
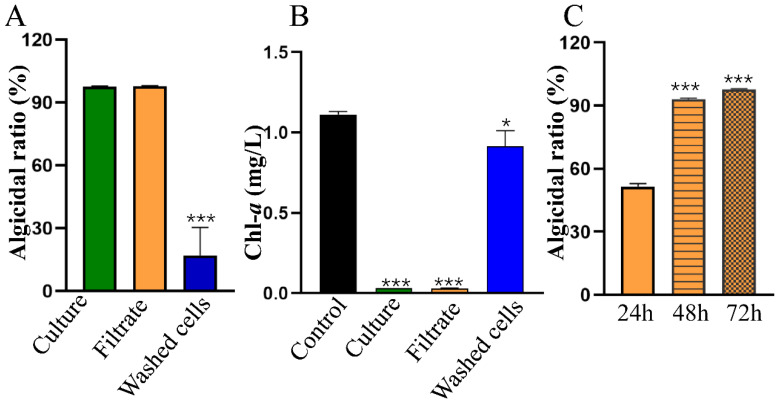
Algicidal modes and ratios of HY against *M. aeruginosa* FACHB-905. Algicidal ratios of bacterial culture, cell-free filtrate, and washed cells of HY against *M. aeruginosa* FACHB 905 at 72 h (**A**). Dynamics of Chl-*a* content (**B**) and algicidal ratios (**C**) of *M. aeruginosa* FACHB 905 after exposure to cell-free filtrate of HY at given time. * *p* < 0.05. *** *p* < 0.001.

As shown in Figure 1D, HY reached the exponential growth phase after 4 h of culture; this phase lasted for 10 h. Several *Streptomyces* spp. strains secrete active compounds to kill cyanobacteria [18,19,23], so we tested the algicidal activity of strain HY during the stationary growth phase at 24 h of fermentation. As indicated in Figure 2A, the algicidal ratio of bacterial culture, the cell-free filtrate, and the washed cells against *M. aeruginosa* FACHB 905 was 97.4%, 97.7%, and 16.97%, respectively, on the 3rd day. The *M. aeruginosa* FACHB 905 culture after treatment was yellow-to-white. Almost all cyanobacterial cells were lysed by HY bacterial culture or cell-free supernatant from HY in 3 days, and the content of the photosynthesis pigment Chlorophyll *a* (Chl-*a*) also decreased rapidly from 0.78 mg/L to 0.03 mg/L or 0.028 mg/L (Figure 2B), respectively. The Chl-*a* content of the washed-bacterial-cells-treated group and control group was 0.91 mg/L and 1.11 mg/L, respectively (Figure 2B). The cultures both remained green. These results showed that HY killed *M. aeruginosa* efficiently via an indirect mode by secreting algicidal compounds. Therefore, we used its cell-free filtrate in subsequent studies.

### 2.3. Algicidal Spectrum of HY

To explore the algicidal spectrum of HY, we tested several cyanobacterial strains, as shown in Table 1. HY displayed high algicidal activity against all single cell strains, with an algicidal ratio of 99.78% against *M. aeruginosa* FACHB 526, 95.20% against *M. aeruginosa* PCC 7806, and 88.74 % against *Synechocystis* sp. PCC 6803. HY also exhibited high algicidal activity against the filamentous cyanobacteria tested, with an algicidal ratio of 88.44% for *Pseudoanabaena* sp. WZU 1801, 97.5% for *Dolichospermum* sp. WZU 1811, and 64.97% for *Anabaena* sp. PCC 7120. However, HY showed much lower algicidal effects against the eukaryotic green algae *Scenedesmus obliquus* (*S. obliquus*) (25.74%). These data demonstrated that HY showed broad-spectrum algicidal activity against cyanobacteria, with few side effects on other non-target eukaryotic phytoplankton species. HY may have a great potential for controlling the threat of multiple HCBs, especially *M. aeruginosa*.

### 2.4. Effect of HY on Cell Morphology of M. aeruginosa

**Figure 3:**
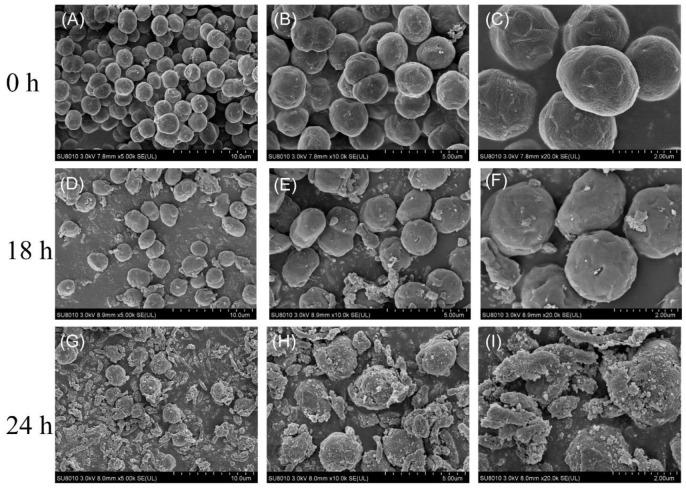
Scanning electron micrographs of *M. aeruginosa* FACHB 905 cells after exposure to *Streptomyces* sp. HY culture filtrate. (**A**–**C**) *M. aeruginosa* cells without HY filtrate treatment at ×5000, ×10,000, and ×20,000, magnification, respectively. (**D**–**F**) and (**G**–**I**) *M. aeruginosa* cells with 18 h and 24 h HY filtrate treatment shown at above magnification, respectively.

To further elucidate the algicidal effects of HY against cyanobacterial cells, we used the typical MC-producing *M. aeruginosa* strain FACHB 905 for further studies. *M. aeruginosa* FACHB 905 was exposed to the cell-free filtrate of HY for 18 h and 24 h, and cell morphological changes were observed by SEM. As Figure 3A–C shows, *M. aeruginosa* cells from the control group were round and retained an intact cell surface. In contrast, most of the algal cells became shrunken and deformed after 18 h of treatment with HY cell-free filtrate, and some algal cells began to break down (Figure 3D–F). As the treatment time lengthened, most cells were lysed, leaving just a few intact *M. aeruginosa* cells with a rough surface and lots of cell debris at 24 h (Figure 3G–I). The cyanobacterial cell walls were severely damaged, leading to the leakage of intracellular components. A similar phenomenon was observed using a light microscope. Our data clearly suggested that HY killed algal cells through indirect attack.

### 2.5. Effect of HY Filtrate on the Content of ROS and MDA and the Activity of SOD

**Figure 4:**
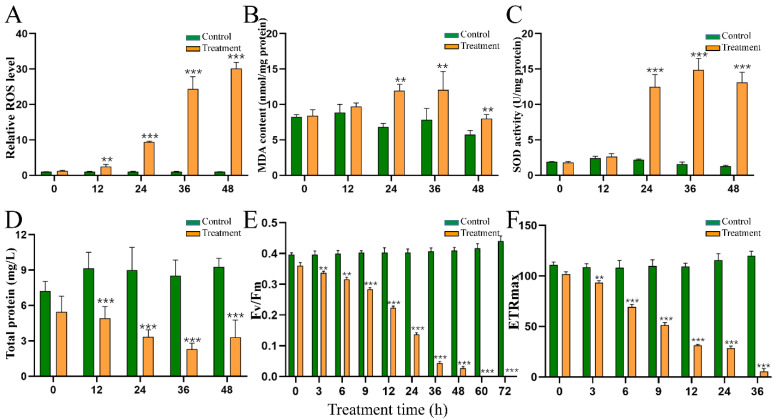
Dynamics of intracellular ROS level (**A**), MDA content (**B**), SOD activity (**C**), total protein (**D**), F_v_/F_m_ (**E**), and ETR_max_ (**F**) of *M.aeruginosa* FACHB 905 cells under the treatment of HY filtrate. ** *p* < 0.01. *** *p* < 0.001.

To study the algicidal mechanisms of HY, we assessed changes in the antioxidant systems of *M. aeruginosa* cells at indicated time points. Intracellular ROS were significantly induced by the supernatant of HY (Figure 4A) when compared with those of the untreated group. Relative levels of ROS increased about 2.50, 9.40, 24.34, and 30.15 times after 12, 24, 36, and 48 h (Figure 4A), respectively, when compared with the control group. The initial MDA content was about 8.41 mol/mg protein in *M. aeruginosa* and remained stable in the control group. The MDA content increased by about 0.76, 0.54, and 0.39 times at 24, 36, and 48 h after HY treatment (Figure 4B), respectively. We also measured the activity of SOD, a typical antioxidant enzyme, to study the response of *M. aeruginosa*. As Figure 4 shows, SOD activity increased over time, and was about 5.78, 9.44, and 10.34 times higher than that of the control group at 24, 36, and 48 h (Figure 4C), respectively. It is worth noting that although SOD showed high activity, algicidal compounds in HY supernatant still induced massive increases in ROS and MDA, resulting in oxidative stress in *M. aeruginosa* cells. The content of total protein in *M. aeruginosa* declined obviously (Figure 4D).

### 2.6. Effect of HY Filtrate on the Photosynthesis System of M. aeruginosa

To further explore the physiological response of *M. aeruginosa* to HY filtrate, we monitored F_v_/F_m_ and ETR_max_. The F_v_/F_m_ (Figure 4E) and ETR_max_ (Figure 4F) of *M. aeruginosa* declined rapidly following treatment with HY supernatant. The F_v_/F_m_ value decreased from 0.36 to 0.22, 0.14, 0.043 (12.04% of the initial value), and 0.026 (7.41% of the initial value) at 12 h, 24 h, 36 h, and 48 h, respectively. Finally, the F_v_/F_m_ value declined to 0 at 60 h. Similarly, the ETR_max_ value of treated *M. aeruginosa* cells decreased dramatically when compared with that of the control group, from 102 to 69.2, 31.33, 28.53, and 5.5 at 6 h, 12 h, 24 h, and 36 h, respectively.

### 2.7. Effect of HY Culture on the Content and Distribution of MC-LR

**Figure 5:**
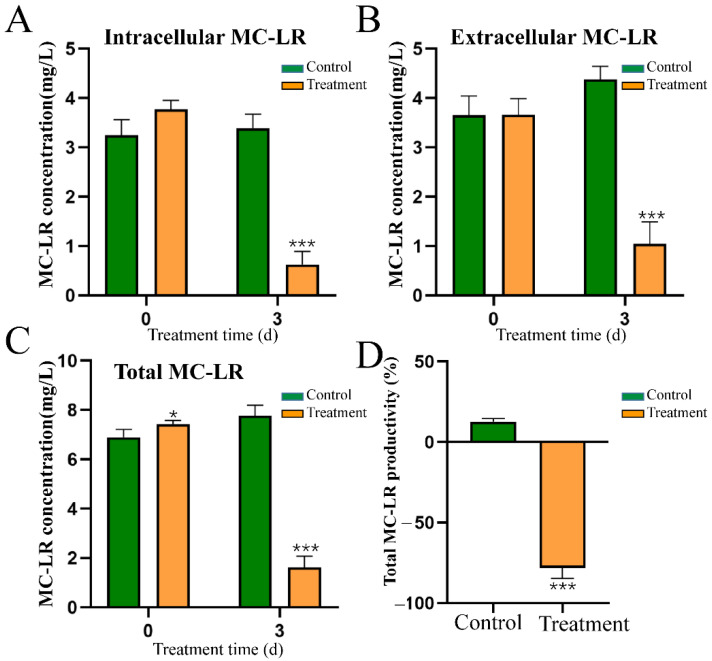
Effect of HY culture on the intracellular (**A**), extracellular (**B**), total MC-LR concentration (**C**), and MC-LR productivity (**D**) of *M. aeruginosa* FACHB 905 during the algicidal process. * *p* < 0.05. *** *p* < 0.001.

Following exposure to HY culture for 3 days, the total content of MC-LR decreased significantly, from 7.77 ng/mL to 1.62 ng/mL (Figure 5C). The intracellular MC-LR concentration of *M. aeruginosa* also declined rapidly (from 3.39 ng/mL to 1.04 ng/mL), which was lower than that of the control group (3.39 ng/mL) (Figure 5A). In addition, the extracellular MC-LR concentration of *M. aeruginosa* cells (0.58 ng/mL) was also significantly lower than that of the control group (4.38 ng/mL) (Figure 5B). Similar to a previous report [23], we found that the MC-LR productivity of the control group increased by 12.67%, when compared with the initial concentration. The MC-LR productivity of the treated groups decreased by 78.22% (Figure 5D). This result suggested that HY not only strongly inhibited the biosynthesis of intracellular MC-LR, but might also be simultaneously involved in extracellular MC-LR degradation.

### 2.8. Effect of HY Filtrate on the Transcription of Key Genes in M. aeruginosa

**Figure 6:**
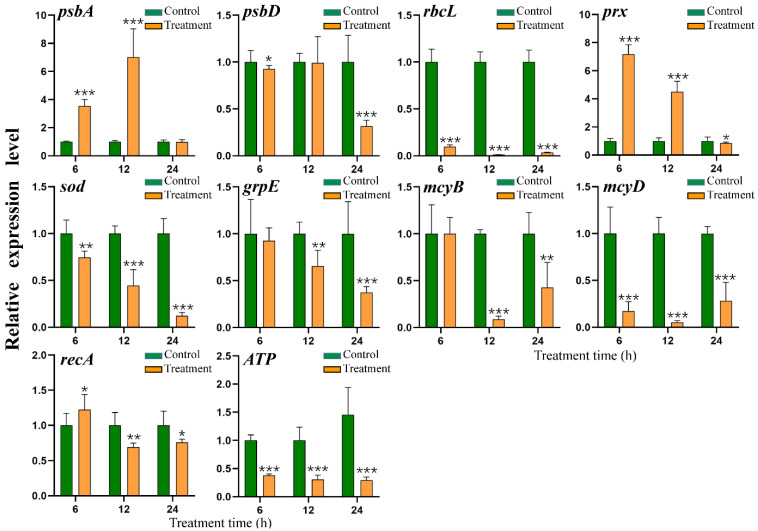
Relative expression level of *psbA*, *psbD*, *rbcL*, *prx*, *sod*, *grpE*, *mcyB*, *mcyD*, *recA*, *ATP* of *M. aeruginosa* FACHB 905 after exposure to the cell-free filtrate of HY for 6 h, 12 h, and 24 h. * *p* < 0.05. ** *p* < 0.01. *** *p* < 0.001.

To further study the mechanism by which HY damages *M. aeruginosa*, we assessed the transcription levels of several key genes (*psbA*, *psbD*, *rbcL*, *prx*, *sod*, *grpE*, *mcyB*, *mcyD*, *recA*, and *ATP*) by RT-qPCR. The *psbA* and *psbD* are important genes encoding the D1 and D2 proteins of photosystem II (PS II), respectively. The expression of *psbA* was increased by approximately 353% and 703% at 6 h and 12 h after treatment with HY supernatant, respectively, finally falling back to a similar level (98.1%) to that of the untreated group after 24 h (Figure 6). The expression of *psbD* did not change very much during the early period of treatment, but decreased by about 30% after 24 h (Figure 6). The *rbcL* gene encodes an important enzyme (RuBisCo) for carbon dioxide assimilation, and its expression pattern was very different from those of *psbA* and *psbD*. *rbcL* expression was significantly inhibited by HY filtrate over time, decreasing to 8.44%, 1.06%, and 3.22% that of the control group expression at 6 h, 12 h, and 24 h, respectively (Figure 6).

The expression patterns of two genes related to antioxidant defense, *prx* and *sod*, were also different from those of *psbA* and *psbD*. *prx* expression was upregulated by 9.98- and 4.5-fold at 6 h and 12 h, respectively, and downregulated 0.16-fold at 24 h. *sod* expression was totally suppressed, decreasing to 75.6%, 44.5%, and 12.3% of the level in the control at 6 h, 12 h, and 24 h, respectively. The transcription levels of two microcystin biosynthesis genes (*mcyB*, *mcyD*) were significantly downregulated at 12 and 24 h, with *mcyD* expression suppressed from the beginning of the treatment period to the end (Figure 6). This result further confirmed that HY efficiently inhibited the biosynthesis of MC-LR in *M. aeruginosa*. The expression of the *recA* gene related to DNA repair was slightly upregulated (22.5%) at 6 h, but then decreased to 30.7% and 24% of the control group at 12 h and 24 h, respectively. Another stress response gene, *grpE*, shared a similar expression tendency, with expression declining significantly from 12 h to 24 h. Moreover, the expression of the key energy metabolism gene *ATP*, encoding a subunit of ATP synthase, was strongly inhibited by HY filtrate at all timepoints tested, being reduced by approximately 62.14%, 69.29%, and 79.65% at 6 h, 12 h, and 24 h, respectively. This result suggested that HY adversely affected various biological processes of *M. aeruginosa* in a complex manner.

### 2.9. Effect of HY Filtrate on Colonies of M. aeruginosa from Natural Bloom Samples

**Figure 7:**
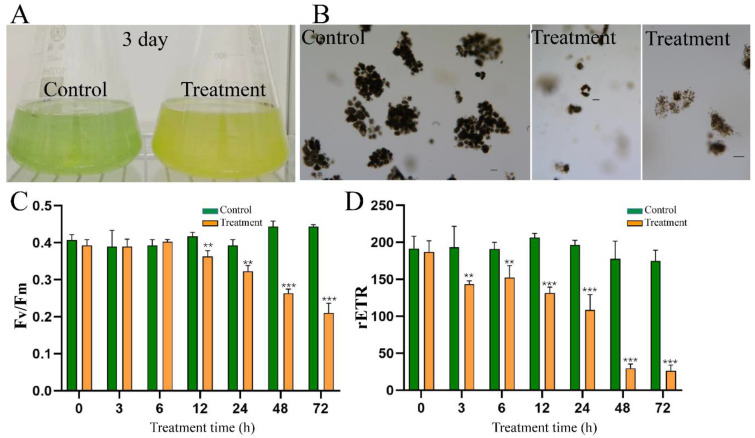
Effect of HY cell-free filtrate on the colonial *M. aeruginosa* from natural bloom body (**A**) and samples observed by light microscope after treated with cell-free filtrate of HY at an inoculum of 5% (*v*/*v*) for 3 days (**B**). Dynamics of F_v_/F_m_ (**C**) and ETR_max_ (**D**) of colonial *M. aeruginosa* cells under the treatment of HY filtrate. ** *p* < 0.01. *** *p* < 0.001. Bar = 50 μm.

Previous studies have demonstrated that algicidal bacteria could easily kill single cells of *M. aeruginosa*, but may not be active against colonial *M. aeruginosa* from natural waters [16,24]. We, therefore, investigated the algicidal activity of HY against *M. aeruginosa* colonies from natural bloom samples of the Xianju Reservoir. Treatment with HY filtrate efficiently flocculated colonial *M. aeruginosa* to the bottom of culture vessels and induced a fast decline of F_v_/F_m_ and ETR_max_ (Figure 7C,D). However, the removal rate of HY filtrate was only 51.77% at 72 h, significantly lower than that observed in *M. aeruginosa* cultures (Figure 7A,B). These results suggested that algicidal compounds from HY could not efficiently lysate colonial *M. aeruginosa* and should be studied further before being used to control *M. aeruginosa* blooms in natural water bodies.

## 3. Discussion

Water eutrophication leads to the occurrence of HCBs worldwide. *Microcystis* is a widespread, bloom-forming, and toxic cyanobacterial group. Many efforts have been devoted to developing novel technologies to control HCBs, and the usage of algicidal bacteria has shown promising application potential. In this study, we aimed to isolate novel algicidal bacterial species and develop effective biological treatments against HCBs. *Streptomyces* sp. HY with high algicidal activity was successfully identified (Figure 1). Strain HY induced 93.04% lysis of *Microcystis* cells within 2 days and almost 100% lysis within 3 days (Figure 2). Several algicidal strains with activity against cells of *Microcystis* spp. have been reported, such as *Streptomyces* sp. L74 [19], *Streptomyces globisporus* G9 [24], and *Streptomyces* sp. HG-16 [23], which can lyse more than 70% *M. aeruginosa* cells within 5 days. However, the highest average algicidal activity was 94.6% by *S. globisporus* G9 in 5 days of treatment. The algicidal activity (97.72%) of *Streptomyces* sp. HY in 3 days was much higher than most of the bacterial strains reported previously. Therefore, *Streptomyces* sp. HY is a promising candidate as an algicidal bacterial strain for controlling HCBs.

Considering that most algicidal bacteria showed obvious host-specificity and only worked well against a few species [20], we tested the lytic spectrum of *Streptomyces* sp. HY. We first assessed its regional host susceptibility using various *Microcystis* spp. strains. For instance, *M. aeruginosa* FACHB 905 (MC-producing) and *M. aeruginosa* FACHB 526 (non-MC-producing) are from China, whereas *M. aeruginosa* PCC 7806 (MC-producing) is from Europe. HY could lyse more than 91% of cells of each *M. aeruginosa* strain within 3 days, regardless of their origin (Figure 2 and Table 1). Several previous studies suggested that MCs might protect *M. aeruginosa* against algicidal substances and diverse allelopathic materials [25,26]. However, the removal efficiency of HY for each *M. aeruginosa* strain was almost the same (>95%) in 3 days, and none of the strains could withstand the damage caused by HY (Table 1) in this study. Therefore, *Streptomyces* sp. HY had a broad host spectrum against *M. aeruginosa* and might work well at a large geographic scale. However, colonial *Microcystis* samples from naturally blooming water bodies seem more resistant to HY supernatant than pure cultures in the lab (Figure 7). Most other strains reported share this limitation [16], except for *Xanthobacter autotrophicus* HYS0201-SM02 [27]. Algicidal bacteria, including HY, should, therefore, be used with chemical reagents or physical equipment to disrupt the colonial state of *Microcystis*. Alternatively, increasing the inoculum ratio of algicidal bacteria might also work well against environmental strains, and further studies should be conducted.

We also tested the algacidal activity of *Streptomyces* sp. HY against other dominant species of bloom-forming cyanobacteria (Cyanophyta), including *Dolichospermum*, *Anabaena*, and *Pseudoanabaena*. *Streptomyces* sp. HY effectively lysed *Dolichospermum* (97.50%), *Anabaena* (64.97%), and *Pseudoanabaena* (88.44%) in 3 days, respectively (Table 1). Interestingly, HY had a minor impact on eukaryotic microalgae, such as *S*. *obliquus* (Chlorophyta) (Table 1) only killing about 25.74% within 3 days. Our results showed that HY was host (cyanobacteria)-specific and had few side effects on other non-target phytoplankton in the natural aquatic environment (Table 1), suggesting great potential for controlling HCBs. The lower sensitivity of *S. obliquus* to HY may be due to intrinsic differences in cellular structure between Chlorophyta and Cyanophyta.

Most algicidal bacterial strains (about 70%) work via indirect attack by secreting active algicidal compounds [20]. To study the algicidal mode of *Streptomyces* sp. HY, we analyzed the algicidal rate of its bacterial culture, cell-free supernatant, and washed cells, respectively. The filtrate of HY showed similar algicidal activity to the bacterial culture against *M. aeruginosa* (97.72% vs. 97.70%) within 3 days, whereas the washed cells exhibited much lower algicidal activity (16.97%) (Figure 2). This indicated that HY excreted substances into the medium to lyse *M. aeruginosa* cells via indirect attack. We also noted that algicidal substances from HY could eventually damage the cellular structure of *M. aeruginosa* cells (Figure 3). The result is in line with previous studies [23,28] The potential anti-cyanobacterial substances produced by HY are unknown, but we speculate that they are amino acid derivatives or other small molecules (unpublished data). These compounds produced by HY will be further isolated and characterized to identify their molecular structures, with the hope of mass-producing them for the ecofriendly control of HCBs.

F_v_/F_m_ and ETR_max_ reflect the photosynthetic activity of cyanobacteria. Therefore, we monitored changes of these parameters during HY treatment. The value of F_v_/F_m_ reduced significantly from 3 h after treatment and decreased to 0 at 60 h (Figure 4E). This decrease suggested that the photosystem of *M. aeruginosa*, especially PS II, was seriously damaged and led to the inhibition of photosynthesis. Additionally, the change in ETR_max_ showed a similar pattern. Its value was reduced significantly at 3 h after treatment and decreased to 3.9% that of the control group at 36 h (Figure 4F). This result suggested that the electron transport chain was seriously blocked. Moreover, the content of Chl-*a* was reduced from 1.11 mg/L to 0.003 mg/L after 72 h of treatment (Figure 2). We noted that the values of F_v_/F_m_ and ETR_max_ were significantly downregulated at 12 h, much earlier than the death of cyanobacterial cells (Figure 5). Therefore, photosynthetic parameters may be important indicators during large-scale cyanobacteria culture.

We also detected the transcription levels of three key photosynthesis-related genes using qRT-PCR. The *rbcL* gene encodes the large subunit of Rubisco, a rate-limiting enzyme for carbon dioxide (CO_2_) fixation. The transcription of *rbcL* was strongly inhibited by HY treatment, decreasing to 3.22% of the control group after 24 h (Figure 6). The *psbD* gene encodes the D2 protein, a key subunit of the reaction center of PSII. Although *psbD* did not seem to be obviously influenced by substances from HY at 6 h and 12 h, its expression was finally reduced to 68.4% of that of the control at 24 h. The transcriptional suppression of *rbcL* and *psbD* was in line with the decline of photosynthetic parameters, in accordance with previous studies [11,28]. Hence, we speculated that the photosynthetic system was the early and primary target of HY algicidal substances. In our study, the expression of *psbA* (encoding the D1 subunit of PSII) increased 3.52-fold and 7.03-fold during the early phase of HY treatment (6 h and 12 h) and maintained a similar level to the control group after 24 h of treatment (Figure 6). Similar results were also described when *M. aeruginosa* was treatment with either 60 mg L^−1^ succinic acid [29] or algicidal supernatant [23]. The upregulation of *psbA* may be caused by *M. aeruginosa* cells trying to compensate for the inhibition of other photosynthesis-related genes.

Impairment of the photosynthesis system promotes the overproduction of ROS. Unfortunately, the photosynthesis system itself is very susceptible to ROS and oxidative stress [28,30]. This negative cascade loop strongly promotes the accumulation of cellular ROS, which react with biomolecules (including DNA, unsaturated fatty acids of membranes, and proteins) in *M. aeruginosa* cells. Therefore, we measured the contents of ROS and MDA during the algicidal process (Figure 4). We found a marked increase in ROS and MDA contents from 24 h to 48 h (Figure 4), clearly indicating that HY filtrate induces oxidative stress in *M. aeruginosa* cells. This trend was consistent with the increase in SOD activity (Figure 4).

The antioxidant enzymes peroxiredoxin (Prx) and SOD are encoded by *prx* and *sod*, respectively. They both play an important role in maintaining intracellular redox homeostasis by scavenging ROS [28,29]. We observed a marked increase in *prx* expression after treatment with HY supernatant (Figure 6). *sod* expression was totally inhibited, and *prx* and *sod* were downregulated as exposure time lengthened (Figure 6). This process would accelerate the shortage of antioxidant enzymes and trigger serious oxidative stress in *Microcystis* cells. To make matters worse, the transcription of other anti-stress genes, such as *recA*, and *grpE*, was also depressed. RecA plays a critical role in repairing damaged DNA [11,28], whereas GrpE promotes the folding of proteins as a chaperone [11,28]. After 24 h of exposure to HY filtrate, *recA* and *grpE* expression decreased to 76.08% and 37.14%, compared to the control group, respectively (Figure 6). The reduction of *recA* and *grpE* expression indicated that the DNA repair system and protein refolding system were both severely damaged in *M. aeruginosa* cells. Moreover, the transcription of a gene related to ATP biosynthesis was simultaneously suppressed (Figure 6). Transcriptional inhibition of *recA*, *grpE*, and *ATP* genes was similar to previous results [28]. Therefore, active substances produced by HY killed cyanobacterial cells by disrupting several key biological processes, including photosynthesis and protein, carbohydrate, and energy metabolism.

The collapse of *Microcystis* cell walls (Figure 3) resulted in the inevitable release of MCs into the medium. Therefore, we investigated the fate of MCs released from *Microcystis* cells treated with HY culture. Unlike the dramatic increase in total MC concentration after chemical treatment (such as CuSO_4_) [8], treatment with HY culture produced an obvious decrease in total MC-LR (Figure 5). Extracellular and intracellular MC-LR concentrations were reduced by 86.86% and 69.23% (Figure 5), respectively, when compared with the control group. More importantly, HY culture significantly suppressed total MC-LR productivity (−78.22%) (Figure 5), whereas *Microcystis* cells treated with algicidal strain HG16 still retained considerable (≈10%) MC-LR productivity [23]. Our data suggest that compound(s) produced by HY are involved in the inhibition of MC-LR biosynthesis. The results from qRT-PCR experiments were in agreement with this phenomenon. Treatment with HY filtrate significantly downregulated the expression levels of microcystin biosynthesis genes *mycB* and *mcyD* at 12 and 24 h (Figure 6). However, the residual concentration of MC-LR was still approximately 2 mg/L (about 20.8% of the control) in co-culture systems after 3 days (Figure 5), which is still much higher than the healthy guideline for MC-LR concentration suggested by the WHO [6]. Considering that MC-LR can persist for several days in the aquatic environment [31], it would pose a threat of secondary environmental pollution after bloom control. Novel strategies for eliminating *Microcystis* blooms and MC-LR at the same time are, therefore, urgently needed. A combination of algicidal bacteria and toxin-degrading bacteria seems simple and feasible [32]. We once isolated a MC toxin degrading strain *Novosphingobium* sp. THN1 with a *mlr* gene cluster from Taihu Lake [33]. Strains HY and THN1 could be used simultaneously against HCBs, and this method is worth further study. Moreover, as the pool of algicidal bacteria expands over time, the option of completely removing HCBs and cyanotoxins simultaneously with one isolate may be possible in the future.

## 4. Conclusions

In conclusion, *Streptomyces* sp. HY exhibited high algicidal activity against *M. aeruginosa* and other harmful cyanobacteria by secreting active compounds, and its algicidal activity was much higher those previously reported strains in the genus of *Streptomyces.* The underlying mechanisms involved impairment of photosynthetic systems, damage to the cellular membrane and cell walls, and oxidative stress. *Streptomyces* sp. HY also inhibited the biosynthesis and release of MC-LR. Our results collectively suggested that *Streptomyces* sp. HY is a promising algicidal strain for the control of HCBs.

## 5. Materials and Methods

### 5.1. Algal Strains and Culture Conditions

*M. aeruginosa* FACHB 905 (MC-producing), *M. aeruginosa* FACHB 7806 (MC-producing), *M. aeruginosa* FACHB 526 (non-MC-producing), *Scenedesmus obliquus* (*S*. *obliquus*) were supplied by the Freshwate Algal Culture Collection at the Institute of Hydrobiology, Chinese Academy of Sciences. *Synechocystis* sp. PCC 6803 and *Anabaena* sp. PCC 7120 were kindly provided by Prof. Xudong Xu (Institute of Hydrobiology, Chinese Academy of Sciences). *Pseudoanabaena* sp. WZU 1801 and *Dolichospermum* sp. WZU 1811 were maintained in our lab. All strains used in this study were cultivated in sterile BG11 medium (components are listed in Table S1) at 25 ± 0.5 °C with a 12 h/12 h (light and dark) cycle and 2500 lux of light intensity. Algae cultures were manually shaken every 12 h, and exponential cells with a starting density of 5 × 10^6^ cells/mL were used in this study.

### 5.2. Screening and Identification of Algicidal Bacteria

Soil samples were collected from Zhejiang and Fujian Provinces (China) in November 2021. Soil samples were placed into a sterilized bottle with 90 mL water and glass beads on a rotary shaker (28 °C, 180 rpm for 1 h). Following the screening method described by [28], a strain #332 (HY) with high algicidal activity was screened from 412 candidates and selected for subsequent investigation. Each single colony was obtained by re-streak on R2A medium (components are listed in Table S2) agar plates, and all strains were preserved at −80 °C in R2A medium containing 20% glycerol in our lab, respectively. Bacterial cell density was quantified by plating a dilution series onto R2A agar plates. To identify the algicidal bacteria strain #332, genomic DNA was extracted by using FastDNA Spin Kit for Soil (MP Biomedicals, Santa Ana, CA, USA), and the bacterial 16S ribosomal RNA gene was amplified by two universal primers, 27F (5′-AGAGTTTGATCATGGCTCAG-3′) and 1494R (5′-GGTTACCTTGTTACFACTT-3′). PCR products were sequenced in a biotechnology company. The sequence had been deposited in the GenBank/EMBL/DDBJ (accession number SUB12519787). The phylogenetic tree was constructed by a MAGE 11.0 software with the Neighbor-Joining method.

### 5.3. Determination of Algicidal Mode, Activity and Spectrum

To explore the algicidal activity and mode of HY, a single colony was inoculated into 50 mL R2A liquid medium, and incubated for 24 h (28 °C, 180 rpm) to the stationary period. Then, the culture was used to prepare supernatant and precipitated cells by centrifugation (12,000 rpm, 4 °C, 5 min). The supernatant was filtered through a 0.45 μm membrane (Millipore, Burlington, MA, USA) to prepare a cell-free filtrate. The remaining pellets were washed with BG11 medium twice, and lastly, re-suspended with the same volume of BG11 medium. Either the bacterial culture, washed cells, or cell-free filtrate were added with a dose of 5% (*v*/*v*) to the *M. aeruginosa* culture (5 × 10^6^ cells/mL), and an equal volume of R2A medium was added into the control group. Each flask was shaken every 12 h manually, and sampled (1.0 mL) every 24 h. Samples (1 mL) were fixed with 0.1 mL Lugol’s solution, and then *M. aeruginosa* cells were counted by a hemocytometer under an optical microscope (Leica, German). The algicidal rate of strain HY against *M. aeruginosa* was calculated with the following formula: algicidal rate (%) = (C_0_ − C_t_)/C_0_ × 100% [28]. C_0_ represents the *M. aeruginosa* cell density of the control group, and C_t_ represents the *M. aeruginosa* cell density of the treated group. To study the algicidal spectrum of HY, cell-free filtrate of HY was added into the tested cyanobacterial cultures (5%, *v*/*v*), and R2A medium was used as the control. *M. aeruginosa* FACHB 905, FACHB 7806, FACHB 526, *Synechocystis* sp. PCC 6803, *Anabaena* sp. PCC 7120, *Pseudoanabaena* sp. WZU 1801, *Dolichospermum* sp. WZU 1811, and *S. obliquus* (initial cell density 5 × 10^6^ cells/mL) were tested. Cyanobacterial cell number was counted after 3 days.

### 5.4. Determination of Chlorophyll a Concentration

Samples were collected by centrifugation (4 °C, 8000 rpm, 5 min) and then immersed in acetone without light at 4 °C to extract Chl-*a*. The absorption of Chl-*a* was detected at 663 and 645 nm with a spectrophotometer and calculated with the following formula [11,34]:

Chl-*a* (mg/L) = 12.72 × OD_663_ − 2.7 × OD_645_

### 5.5. Determination of F_v_/F_m_ and ETR_max_

The relative maximum quantum yield (F_v_/F_m_) and the maximum photo synthetic rate (ETR_max_) of Photosystem II (PS II) were measured at indicated time point by a pulse-amplitude-modulated fluorometer (PHYTO-PAM-II, WALZ, Effeltrich, Germany), as our previous study described [28]. Each sample was kept in dark conditions for 3–8 min before the detection.

### 5.6. Analyses of Intracellular ROS Level

The fluorescent dye 2′,7′-dichloro-dihydro-fluorescein diacetate (DCFH-DA) (Beyotime, Shanghai, China) was used to detect the intracellular ROS levels of *M. aeruginosa* cells, as with our previous studies [28,35]. Briefly, algal cells were treated with cell-free filtrate (5%, *v*/*v*) for 12, 24, 36, and 48 h. Then, cells were harvested at indicated time point and incubated with 10 μM DCFH-DA for 30 min at 25 °C in dark. *M. aeruginosa* cells were then washed with PBS three times to remove extracellular DCFH-DA probe. Finally, the fluorescence intensity (representing the ROS level) was detected using a microplate reader (Molecular Device, San Jose, CA SpectraMax iD3, USA). The excitation and emission wavelengths were 488 and 525 nm, respectively.

### 5.7. Content of MDA and Activity of SOD

*M. aeruginosa* was treated with HY cell-free filtrate (5%, *v*/*v*), and cells from 10 mL sample were collected at indicated time point day by centrifugation (4 °C, 5000 rpm, 5 min). Then, *M. aeruginosa* cells were disrupted by a cell disruptor (Life Real Biotechnology Co., Ltd., Hangzhou, China) for 10 min (6.5 m/s, ultrasonic time 1 min; interval time: 1 min) at 4 °C in PBS. The supernatant was obtained by centrifugation (12,000 rpm, 10 min, 4 °C), and was used for evaluation the activity of SOD and content of MDA with corresponding kits (Beyotime, Shanghai, China).

### 5.8. Observation of the Cellular Morphology by Scanning Electron Microscope (SEM)

*M. aeruginosa* cells were treated with 5% cell-free filtrate of HY for 0 h, 18 h, or 24 h, and collected by centrifugation (4 °C, 8000 rpm, 5 min). *M. aeruginosa* cells were fixed 12 h in 0.1 M phosphate-buffered saline (PBS) containing 2.5% glutaraldehyde solution (*v*/*v*). Next, cells were washed with 0.1 M PBS 3 times, and treated by 1% osmic acid solution for 1–2 h. Finally, *M. aeruginosa* cells were dehydrated in different concentration of ethanol solution, and were examined by SEM (JEOL, JSM-6510LV, Tokyo, Japan) after drying and coating with gold.

### 5.9. Analysis of MC-LR

*M. aeruginosa* was co-cultivated with HY (5%, *v*/*v*) for 3 days, and cells from 4 mL sample were collected at day 0 and the 3rd day by centrifugation (4 °C, 5000 rpm, 5 min). The cell-free filtrate was used for detection of the extracellular MC-LR, whereas the left cells were broken by a cell disruptor at 4 °C (Life Real Biotechnology Co., Ltd., Hangzhou, China) in PBS buffer. The supernatant after centrifugation (4 °C, 5000 rpm, 5 min) was used for measuring the content of intracellular MC-LR with an enzyme-linked immunosorbent assay (ELISA) kit from the Institute of Hydrobiology (Chinese Academy of Sciences, Wuhan, China). The MC-LR productivity was calculated as the previous study described [23].

### 5.10. Effect of HY on Filed Blooms Dominated by Microcystis spp.

Natural bloom samples, mainly dominated by colonial *M. aeruginosa*, and some colonies of *M. novacikii* and *M. flos-aquae* (about 99.47% in cyanobacterial population) were collected from the Xianju Reservoir, Wenzhou, Zhejiang, China (27°56′48.9″ N, 119°78′61.9″ E) on 25 June 2022. These bloom samples were transferred into flasks within 2 h, and diluted 4 times by sterile BG11 medium the next day. The cell-free filtrate of the strain HY culture was added to algal cultures at 5% (*v*/*v*). Samples were observed under a light microscope (DMi5, Leica Biosystems), and algacidal activity, F_v_/F_m_, and ETR_max_ were also evaluated as previously described [28].

### 5.11. Effect of Strain HY on the Key Gene Expression of M. aeruginosa

To probe the underlying algicidal mechanisms of HY against *M. aeruginosa*, the transcriptional levels of several key genes related to photosynthesis, MCs biosynthesis, anti-oxidation, and DNA repair were detected. *M. aeruginosa* samples were treated by cell-free supernatant of HY (5%, *v*/*v*) for 6, 12, and 24 h, and then harvested by centrifugation. Subsequently, total RNA was extracted by using Trizol reagent (Invitrogen, Carlsbad, CA, USA) [36]. The elimination of residual genomic DNA and reverse transcription used the PrimeScript™ RT reagent kit containing gDNA Eraser (Takara, Beijing, China). First-strand cDNA was generated from 1 g of total RNA, and quantitative real-time PCR (qRT-PCR) was performed with a Q1 Real-Time PCR System (Applied Biosystems, Waltham, MA, USA). The 15 μL of reaction mixture contained 7.5 μL of the Power SYBR^®^Green PCR master mixture (Life Technology, New York, NY, USA), 0.45 μL of each primer (final concentration is 10 pmol), 1.5 μL of cDNA template, and 5.1 μL nuclease-free water. The following amplification program was 1 cycle of 50 °C for 2 min, a secondary cycle of 95 °C for 10 min, following by 40 cycles of 95 °C for 15 s, and 60 °C for 1 min. The primers for target genes used in this study are listed in Table 2. The 2^−△△Ct^ method was used to determine the transcription level of each target gene [37], and *16S* rRNA was chosen as the internal reference gene.

### 5.12. Statistical Analysis

Each group in this study has three independent repetitions, and all data are presented as means ± SD unless stated. SPSS 18.0 software (SPSS Inc., Chicago, IL, USA) was used for statistical analysis. Significant difference level was considered when *p* < 0.05.

## Figures and Tables

**Table 1:** Algicidal effect of strain HY against various cyanobacterial strains.

Strain	Algicidal Ratio (%)
*Microcystis aeruginosa* FACHB 526	99.78 ± 0.00
*Microcystis aeruginosa* PCC 7806	95.20 ± 0.01
*Synechocystis* sp. PCC 6803	88.74 ± 0.01
*Pseudoanabaena* sp. WZU 1801	88.44 ± 0.00
*Dolichospermum* sp. WZU 1811	97.50 ± 0.00
*Anabaena* sp. PCC 7120	64.97 ± 0.06
*Scenedesmus obliquuso*	25.74 ± 0.02

**Table 2:** Primer sequences for real-time quantitative PCR.

Gene	Forward Primer (5′-3′)	Reverse Primer (5′-3′)
*psbA*	GATCCAAGGACGCATTCCTA	CAACGGTGGTCCTTACCAG
*psbD1*	TCGCAGTGACCATGGAGTAG	TTGAAGACGGTGAAGGTT
*rbcL*	CGTTTCCCCGTCGCTTT	CCGAGTTTGGGTTTGATGGT
*prx*	GCGAATTTAGCAGTATCAACACC	GCGGTGCTGATTTCTTTTTTC
*sod*	GAACCAACCAAGCCCAACC	CAACAATGCCGCCCAAG
*grpE*	CGCAAACGCACAGCCAAGGAA	GTGAATACCCATCTCGCCATC
*mcyB*	CCTACCGAGCGCTTGGG	GAAAATCCCCTAAAGATTCCTGAGT
*mcyD*	ACCCGGAACGGTCATAAATTGG	CGGCTAATCTCTCCAAAACATTGC
*recA*	GCCGAACAAACTAACGTGGT	GGAACCGAACCCATGTC
*ATP*	GTATGGATATCGTGGACACCG	AGATCGACTAATTTGGGAGCG
*16S*	GGACGGGTGAGTAACGCGTA	CCCATTGCGGAAAATTCCCC

## Data Availability

The data presented in this study are available in the article.

## Supplementary Materials

The following supporting information can be downloaded at: https://www.mdpi.com/article/10.3390/toxins15030220/s1, Table S1: Components of BG11 medium; Table S2: Components of R2A liquid medium.
