# Supplementary material for: Discovery of a High-Efficient Algicidal Bacterium against Microcystis aeruginosa Based on Examinations toward Culture Strains and Natural Bloom Samples
Source: Toxins (Basel). 2023 Mar 14;15(3):220. doi: 10.3390/toxins15030220 (PMC10058357; doi:10.3390/toxins15030220)
Supplement: Supplementary file 1 [file toxins-15-00220-s001.zip › toxins-2255308-supplementary.pdf]

Table S1 Components of BG11 medium

Components	concentration (g/L)
$NaNO_3$	1.5
$MgSO_4 \cdot 7H_2O$	0.075
Citric acid	0.006
$EDTANa_2$	0.001
A5 solution	1ml
K_2HPO_4	0.04
$CaCl_2 \cdot 2H_2O$	0.036
Ferric ammonium citrate	0.006
Na_2CO_3	0.02

A5 solution

Components	concentration (g/L)
H_3BO_3	2.86
$ZnSO_4 \cdot 7H_2O$	0.22
$CuSO_4 \cdot 5H_2O$	0.079
$MnCl_2 \cdot 4H_2O$	1.86
$Na_2MoO_4 \cdot 2H_2O$	0.039
$Co\ (NO_3)_2 \cdot 6H_2O$	0.05

Table S2 Components of R2A liquid medium

Components	concentration (g/L)
Proteose Peptone	0.25
Starch	0.5
Glucose	0.5
Yeast Extract	0.5
Casein Hydrolysate	0.5
Dipotassium Phosphate	0.3
Sodium Pyruvate	0.3
Magnesium Sulfate Anhydrous	0.1
Bacteriological Agar	15.00
pH	7.2±0.2
